# Ecomorphological Analysis of the Bird Lumbosacral Organ in an Evolutionary Context

**DOI:** 10.1002/jmor.70073

**Published:** 2025-08-23

**Authors:** Idriss Pelletan, Raphaël Cornette, Anick Abourachid

**Affiliations:** ^1^ Muséum National d'Histoire Naturelle CNRS, Mecadev Paris France; ^2^ Institut de Systématique, Evolution, Biodiversité (ISYEB), UMR 7205, CNRS Muséum National d'Histoire Naturelle Paris France

**Keywords:** balance, birds, geometric‐morphometrics, pelvic, phylogeny

## Abstract

Birds possess a unique balance organ, the lumbosacral organ (LSO), located in the lumbosacral region of the synsacrum. This organ surrounds the spinal cord and leaves distinct traces of its size and shape on the endocast of the vertebral canal. To date, many questions about the function of the LSO and its implications in bird biology remain. Here, we investigate whether the shape of the synsacral vertebral canal endocast, influenced by the LSO, is related to locomotor habits, pelvic morphology, and phylogeny. We used 2D and 3D geometric morphometrics to characterise the shape of the digital synsacral vertebral canal cast and to test whether its morphology is indicative of locomotor behaviour and pelvic morphology. We also quantified the phylogenetic signal to determine whether phylogeny has an impact on morphology. Our results suggest that the vertebral canal endocast is shaped by the LSO, particularly in predominantly perching birds, where it is proportionally larger than in other locomotor groups. We also show that the pelvic morphology covaries significantly with the vertebral canal morphology. A proportionally larger LSO corresponds to a shorter, wider pelvis, while a smaller LSO corresponds to a longer, more slender pelvis. Finally, in addition to a strong phylogenetic signal in vertebral canal morphology, we identify allometry, indicating that body size also influences LSO morphology.

## Graphical abstract

Our geometric morphometric analysis of 36 bird species reveals locomotor‐linked adaptations in the lumbosacral organ (LSO). Perching birds show proportionally larger LSOs, while aquatic species exhibit elongated vertebral canals. We demonstrate strong pelvic‐LSO covariation and significant phylogenetic signals, supporting the LSO's role in equilibrium control, particularly in arboreal environments. These findings provide an evolutionary framework for understanding avian sensory systems and highlight the LSO's ecological specialisation.

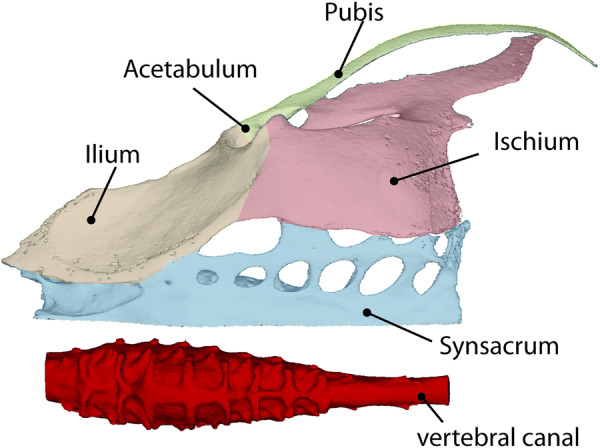

## 1 Introduction

**Figure 1:**
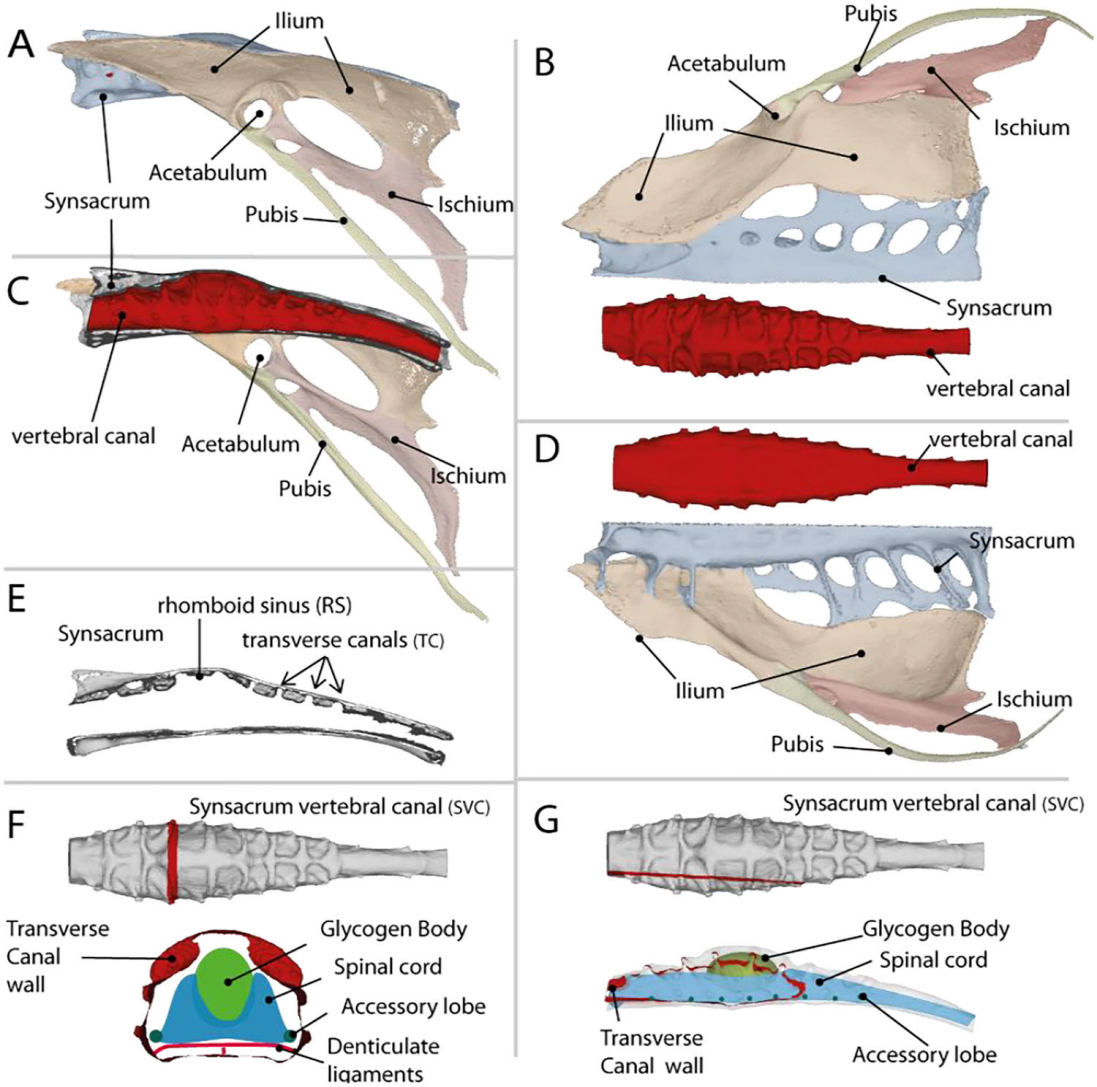
Right part of the pelvic girdle of *Hirundo rustica*. (A) lateral view; (B) dorsal view; (C) medial view; (D) ventral view; (E) transversal view; (F and G) latero‐medial cut and antero‐posterior cut with a schematic representation of the LSO.

Despite their taxonomic and ecological diversity, all birds share a similar body plan (Abourachid and Höfling 2012). One of the features of this body plan is the fusion of the lumbar and sacral vertebrae, forming a long synsacrum to which the pelvis is attached (Figure 1A–D). The canal of the synsacrum houses the spinal cord, which supports sensory processing and motor control (see Stanchak et al. 2020). The spinal cord in the synsacrum is greatly enlarged by an organ unique to birds: the lumbosacral organ (LSO). The shape of the synsacral vertebral canal (SVC) is determined by the LSO, which fills its cavity and leaves an imprint on the internal bone surface, similar to the brain's imprint on the braincase (Kamska et al. 2020; Necker et al. 2000; Stanchak et al. 2020).

The general shape of the LSO can be described as a cylinder whose central part is widened laterally and dorsally. On the upper face, circular arcs are arranged in pairs and oriented perpendicular to the axis of the cylinder. More specifically the LSO can be described as follows: at the level of the rhomboid sinus (RS), the area where the spinal cord is split, the dorsal part of the canal is notably rounded in contrast to its flatter ventral part (Figure 1C). Transverse thinning of the bone forms the transverse canals (TCs) dorsally in the SVC, where the vertebrae fuse. Transverse canals are canals containing spinal fluid and connected to the accessory lobes, making them a structure analogous to the semicircular canals of the inner ear (Necker 2005). The ovoid glycogen body, a cluster of large cells containing glycogen (De Gennaro 1982), is an integral part of the LSO and is integrated dorsally into the spinal cord, creating a separation followed by fusion of the spinal cord at the level of the RS. Ventro‐lateral globular structures, called accessory lobes, are associated with the spinal cord at the level of the TCs. Finally, a network of denticulated ligaments is located ventral to the spinal cord and connects the lateral edges of the SVC (Kamska et al. 2020; Schroeder and Murray 1987).

The LSO has been described since the 19th century (Duval 1877), but hypotheses about its functional role are relatively recent (Necker et al. 2000). The study of the LSO has raised many questions over the years. In particular, it has been hypothesised that the glycogen body making up the LSO could serve as a nutrient reserve (De Gennaro 1961). A study in which the transverse canals were removed without damaging the nerve endings (Necker et al. 2000) showed that the birds had great difficulty maintaining their balance on the ground, but no difficulty in flight. It is now accepted that the LSO plays a role in maintaining balance by acting as a mechanoreceptor organ (Kamska et al. 2020). It thus acts as a second balance management organ in birds, in addition to the inner ear (Biederman‐Thorson and Thorson 1973; Necker 2006).

However, its function is still not fully understood. Taking into account the different components of the LSO and its organisation, as well as the experiments carried out by Necker et al. (2000), four mutually nonexclusive hypotheses of its function have been proposed (Stanchak et al. 2023). The first hypothesis is that the LSO functions in a similar way to the semicircular canals of the inner ear; that is, body movements induce a flow of fluid in the transverse canals, exciting motor neurons in the accessory ganglia. The second hypothesis suggests that the accessory ganglia are excited by the network of denticulated ligaments, acting like a mass accelerometer. The third hypothesis is that motor neurons can detect fluid flow in the spinal canal, amplifying the motor signal. The fourth hypothesis proposes that changes in the physical properties between the various components of the LSO and the spinal cord generate tensions along the latter (Kamska et al. 2020).

Whatever its mode of operation, the LSO is considered a mechanoreceptor organ similar to the inner ear. The inner ear has a considerable morphological diversity (Benson et al. 2017) and morpho‐functional relationships between the vestibular apparatus, locomotor habits and phylogeny have been demonstrated (Ekdale 2016). In mammals, the size, orientation and morphology of the semicircular canals have been correlated with locomotor behaviour, posture and living environment, while retaining a strong phylogenetic signal (Ekdale 2016; Coutier et al. 2017). In birds, correlations between vestibular morphology and locomotor behaviour are less clear (Benson et al. 2017; Knoll and Kawabe 2020). The main constraint on semicircular canal morphology appears to be skull size (Benson et al. 2017; Knoll and Kawabe 2020).

**Figure 2:**
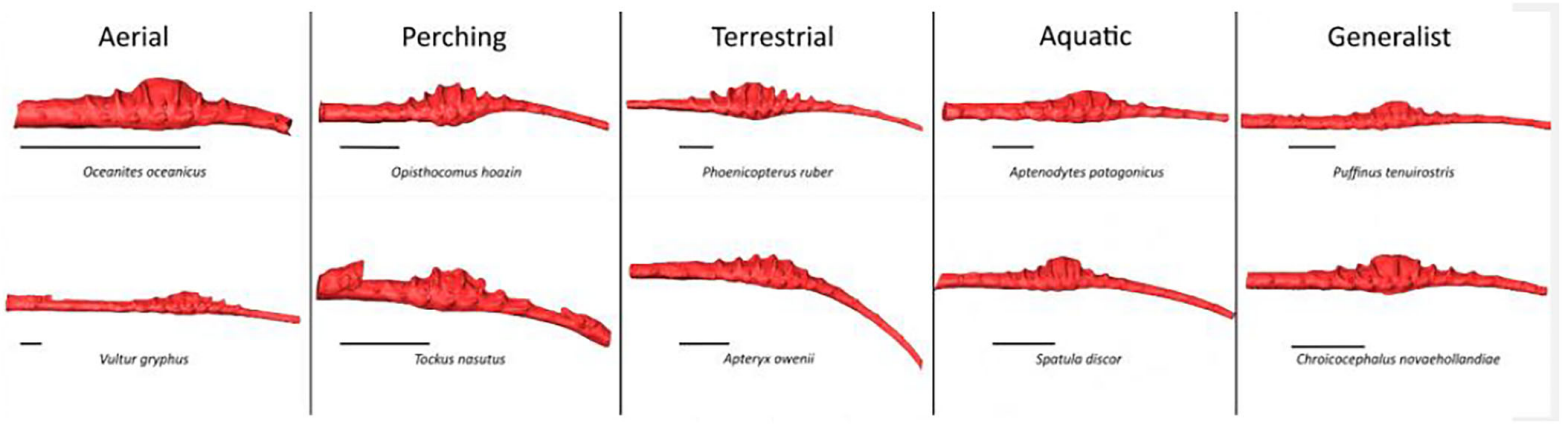
Representation of the morphological diversity of the SVC according to ecological groups.

The morphology of the LSO is also varied and seems to be linked to ecology (Stanchak et al. 2020). In particular, the number and orientation of TCs, as well as the size of the glycogen body, differ among species. Perching birds, for example, have a larger LSO than other species (Figure 2). These birds move through the canopy, a complex environment where the geometry and mechanical properties of branches vary considerably. We therefore hypothesise that there may be a correlation between the morphological variation of the LSO and the environment, and therefore the ecology of a species. As the LSO is incorporated into the sacrum, which fuses with the pelvis, we examined morphological variation in the pelvis, which has previously been shown to correlate with ecological factors (Frank et al. 2022). We then explored the relationship between the LSO and the pelvic structures. Using a geometric morphometrics approach to quantify morphological changes in the pelvis and the SVC endocast, we thus aim to test the relationship between shape, ecology, and phylogeny. We specifically ask: (1) Is the morphology of the endocast of the synsacral vertebral canal, influenced by the LSO, correlated with the locomotor habits of birds? (2) Is there a correlation between the synsacral vertebral canal endocast morphology and pelvic morphology in birds? (3) Is there a phylogenetic signal in the morphological variation of the synsacral vertebral canal endocast? (4) Can an evolutionary allometric signal be detected in the morphological variation of the LSO?

## 2 Materials and Methods

We selected 36 bird species from 33 families and 20 orders, to provide a sample with diverse ecology and phylogenetic relationships (Table 1; reference to museum collection numbers is provided in Supporting Information S3: Table S1). We used 3D models from the Tempo bird database (Bjarnason and Benson 2021) for 35 specimens. One specimen (*Coturnix coturnix*), from the osteological collection of the Muséum national d'Histoire Naturelle in Paris, was scanned using the AST‐RX platform. We used Avizo (4.6) software to create our 3D models of the SVC endocast and synsacrum.

**Table 1:** List of species in this study.

Species	Common name	Sigles	Primary lifestyle	Mass (g)	Species	Common name	Sigles	Primary lifestyle	Mass (g)
*Elanus caeruleus*	Black‐winged kite	Ec	Aerial	259.8	*Opisthocomus hoazin*	Hoatzin	Ho	Perching	696
*Florisuga mellivora*	White‐necked jacobin	Fm	Aerial	7.4	*Probosciger aterrimus*	Palm cockatoo	Pa	Perching	841
*Fregata aquila*	Ascension frigatebird	Fa	Aerial	1473.1	*Pycnonotus cafer*	Red‐vented bulbul	Pc	Perching	42.9
*Hirundo rustica*	Barn swallow	Hr	Aerial	17.9	*Ramphastos ambiguus*	Yellow‐throated toucan	Ra	Perching	651.7
*Oceanites oceanicus*	Wilson's storm petrel	Oo	Aerial	30.4	*Tockus nasutus*	African grey hornbill	Tn	Perching	179.6
*Phaeton lepturus*	White‐tailed tropicbird	Pl	Aerial	328	*Anser fabalis*	Taiga bean goose	Afa	Terrestrial	2754.7
*Sula dactylatra*	Masked booby	Sda	Aerial	1732.1	*Ardea alba*	Great egret	Aa	Terrestrial	871.3
*Vultur gryphus*	Andean condor	Vg	Aerial	11236.1	*Calandrella cinerea*	Red‐capped lark	Cci	Terrestrial	23.7
*Pelecanus occidentalis*	Brown pelican	*Po*	Aerial	*3427.8*	*Columba palumbus*	Common wood pigeon	Cp	Terrestrial	490
*Spatula discors*	Blue‐winged teal	Sdi	Aquatic	359.4	*Coturnix coturnix*	Common quail	Cco	Terrestrial	96.3
*Aptenodytes patagonicus*	King penguin	Ap	Aquatic	11731.1	*Grus leucogeranus*	Siberian crane	Gl	Terrestrial	5913.4
*Aythya ferina*	Common pochard	Afe	Aquatic	823	*Phoenicopterus ruber*	American flamingo	Pr	Terrestrial	3031.6
*Podica senegalensis*	African finfoot	Ps	Aquatic	599	*Picus viridis*	European green woodpecker	Pv	Terrestrial	176
*Chroicocephalus novaehollandiae*	Silver gull	Cn	Generalist	269	*Porphyrio poliocephalus*	Grey‐headed swamphen	Pp	Terrestrial	773.9
*Puffinus tenuirostris*	Short‐tailed shearwater	Pt	Generalist	559	*Recurvirostra avosetta*	Pied avocet	Ra	Terrestrial	304
*Strigops habroptilus*	Kākāpō	Sh	Generalist	1732.1	*Rollulus rouloul*	Crested partridge	Rr	Terrestrial	216.5
*Calyptomena viridis*	Green broadbill	Cv	Perching	58.5	*Turnix varius*	Painted buttonquail	Tv	Terrestrial	90.2
*Coracias benghalensis*	Indian roller	Cb	Perching	157.5	*Apteryx owenii*	Little spotted kiwi	Ao	Terrestrial	1238.3

We classified birds into five main lifestyles using the Avonet database (Tobias et al. 2022), based on their dominant mode of locomotion during foraging: aerial, the species spends most of its time in flight and hunts or feeds mainly in flight; perching, the species spends most of its time perched above the ground, either in the branches of trees or other vegetation; terrestrial, the species spends most of its time on the ground, where it feeds by walking or hopping; aquatic, the species spends most of its time sitting on the water and feeds by floating or diving below the surface of the water; and generalist, the species does not have a primary lifestyle, as it forages in different environments. We used this parameter to infer locomotor behaviour because it defines species according to where they spend most of their time and where they feed (water, ground, air, tree). However, based on the Birds of the World website, Birds of the World—Comprehensive life histories for all bird species and families 2025, we changed the attributions for *Pelecanus occidentalis* from aerial to aquatic, and *Vultur gryphus* from terrestrial to aerial, as we deemed these too different from the main locomotory behaviour.

### 2.1 Phylogenetic Hypothesis

**Figure 3:**
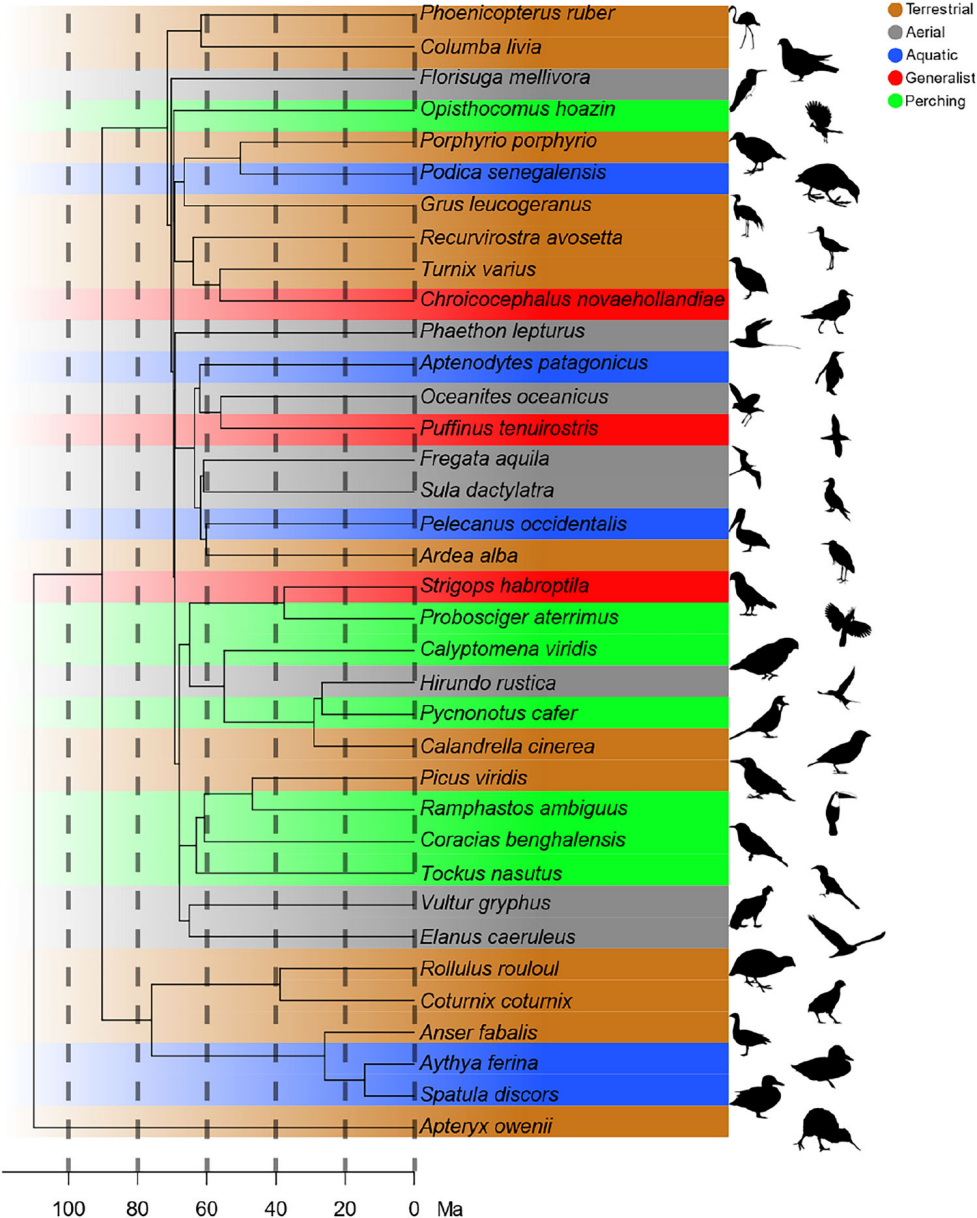
Phylogenetic position of the sample, time‐calibrated tree extracted from Kimball et al. (2019), brown is terrestrial, green is perching, grey is generalist, blue is aquatic, light blue is aerial.

We used the time‐calibrated super‐tree by Kimball et al. (2019) and removed all species not in our data set using the ‘drop.tip’ function of the Ape package (Paradis et al. 2004). If a species in our data set was not listed in this phylogeny, we used a related species instead. If the genus was missing, we used a related genus to represent the family or order. Due to the wide diversity of the orders and families considered, this approach does not significantly influence our results (Figure 3).

### 2.2 Geometric Morphometric Analysis

**Figure 4:**
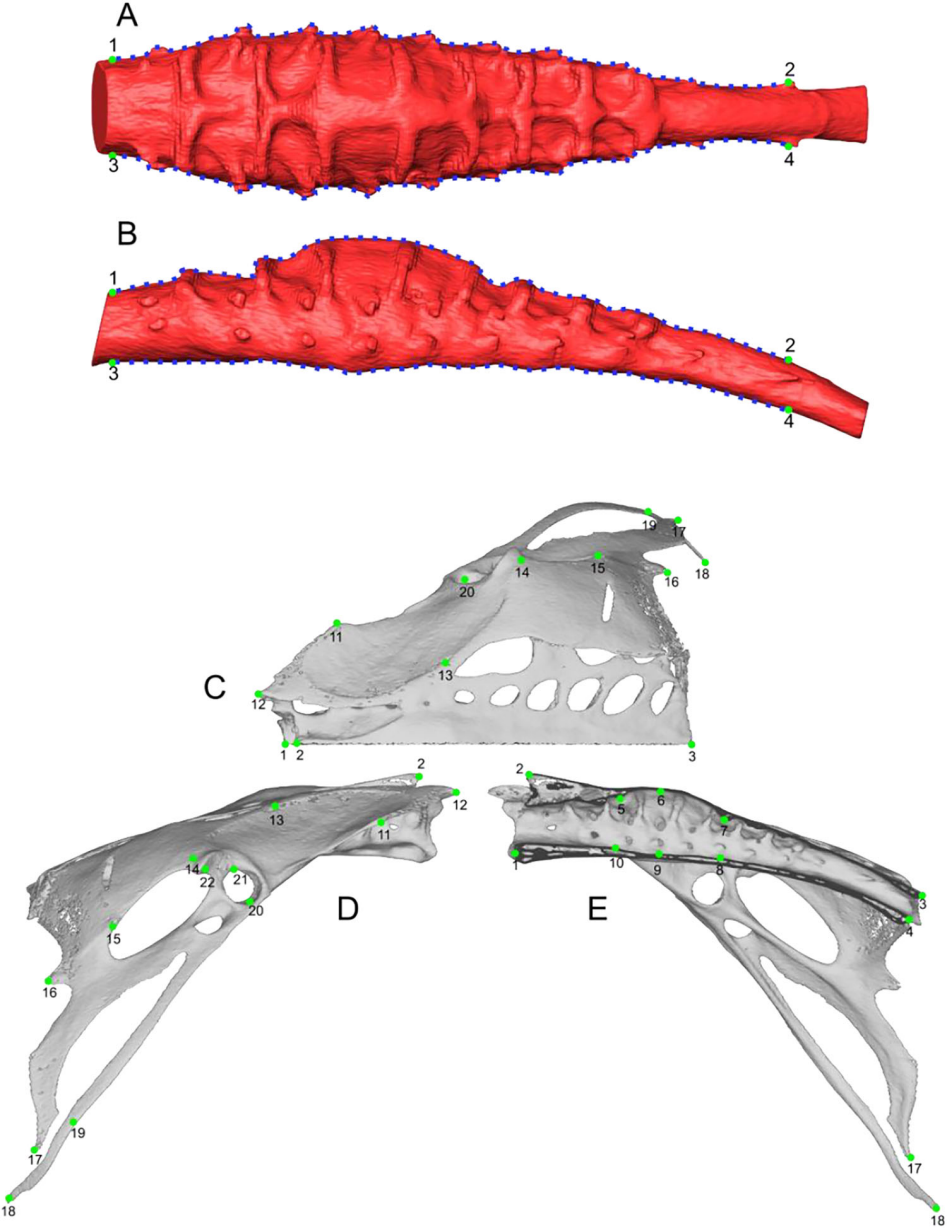
Configuration of 2D landmarks for the LSO in dorsal view (A) and lateral view (B). Configuration of 3D landmarks for the pelvis in dorsal (C), lateral (D) and median (E) views. The green dots correspond to anatomical landmarks; the blue dots correspond to sliding semi‐landmarks, endocast corresponding to *Hirundo rustica*.

We used a 2D geometric morphometric approach to study the shape of the SVC endocast (Zelditch et al. 2004). We used 2D sliding semi‐landmarks, which allow homologous anatomical curves to be defined (Gunz and Mitteroecker 2013), for three main reasons: (i) there are no distinguishable homologous anatomical landmarks between different species, (ii) the combination of lateral and dorsal views provides a robust approximation of the three‐dimensional shape for exploratory analysis, and (iii) the lamellar structure of the LSO, due to TCs, made 3D geometric homology difficult due to the high number of points needed to cover the surface uniformly and instabilities during the sliding phase of the sliding semi‐landmarks. Sliding semi‐landmarks are moved to minimise bending energy between specimens, ensuring geometric homology, (Gunz and Mitteroecker 2013; Rohlf and Slice 1990; Zelditch et al. 2004; see Figure 4). To this end, LSOs were captured from 3D meshes in orthographic and homologous lateral and dorsal views using the Avizo software (4.6). Lumbosacral organs were standardised by aligning the ventral surface of the proximal end horizontally for the lateral view and rotating 90° along the longitudinal axis for the dorsal view. Next, we used TpsDig2 version 2.32 and TpsUtil version 1.82 (Rohlf 2015) to place all landmarks and sliding semi‐landmarks (Table 2). Deformation grids were used to identify the RS, delimited by the beginning and the end of the bulky area in lateral view.

**Table 2:** List of 2D landmarks with their description.

Landmarks n°	Descriptions
1	Most proximal point after the first rib; dorsal for the lateral view; right lateral for the dorsal view
2	Most proximal point after the first rib; ventral for the lateral view; left lateral for the dorsal view
3	Most proximal point before the last rib; dorsal for the lateral view; right lateral for the dorsal view
4	Most proximal point before the last rib; ventral for the lateral view; left lateral for the dorsal view
C1	Curve of 198 sliding semi‐landmarks; dorsal for the lateral view; right lateral for the dorsal view
C2	Curve of 198 sliding semi‐landmarks; ventral for the lateral view; left lateral for the dorsal view

3D geometric morphometric analyses (Zelditch et al. 2012) were performed on pelvic models. To obtain global information about the shape of the pelvis, 16 external anatomical landmarks were defined on the right half of the pelvis (Figure 4C–E). We also incorporated six internal anatomical landmarks (landmarks 5–10, Table 3) into the pelvic landmark set to capture variation in the shape of the synsacral vertebral canal (SVC) at the level of the rhomboid sinus (RS). The software ‘landmarks’ (Landmark version 3.6, Institute for Data Analysis and Visualisation (Wiley et al. 2005) was used to place the points.

**Table 3:** List of 3D landmarks with description as used in this study.

Landmarks n°	Description
1	Medio cranial extremity of the synsacrum, in the centre of the vertebrae centrum
2	Cranio‐dorsal extremity of the synsacrum, in the centre of the neural spine
3	Caudo‐medial extremity of the synsacrum, dorsally
4	Caudo‐medial extremity of the synsacrum, ventrally
5	Medio‐dorsally in the neural canal, cranial part of the rhomboid sinus
6	Medio‐dorsally in the neural canal, at the apex of the rhomboid sinus
7	Medio‐dorsally in the neural canal, at the caudal part of the rhomboid sinus, on the concave end between the TCs
8	Medio‐ventrally in the vertebral canal, ventrally to point 7
9	Medio‐ventrally in the neural canal, ventrally to point 6
10	Medio‐ventrally in the neural canal, ventrally to point 5
11	Lateral end of the cranial part of the preacetabular wing of the ilium
12	Medio‐cranial end of the cranial part of the preacetabular wing of the ilium
13	Iliac crest at the most lateral point of synsacrum (width of synsacrum)
14	Iliac crest at the most lateral point of antitrochanter (width of pelvis)
15	Laterocaudal end of the iliac crest
16	Dorso caudal edge of the ilium
17	Ventro‐caudal end of the ischium
18	Caudal end of the pubis (or as close as possible)
19	Inflection point in line with the caudal part of the ischiopubic window
20	Ventral edge of the acetabulum
21	Dorsal edge of the acetabulum, anterior to the antitrochanter
22	Dorso‐caudal end of the antitrochanter

### 2.3 Statistical Analyses

As species are not independent data points but related by their evolutionary history we used methods that account for phylogeny (Felsenstein 1985). We measured the phylogenetic signal in the shape data using the Kmult statistic (Adams 2014) using the ‘physignal’ function from the geomorph package (Adams and Otárola‐Castillo 2013). Kmult compares the observed rate of morphological variation with the variation expected under Brownian motion to quantify the phylogenetic signal (Adams 2014). When the phylogenetic signal was strong, we performed phylogenetic principal component analyses (pPCA) (Revell 2009) using the ‘gm.prcomp’ tool in the geomorph package (Adams and Otárola‐Castillo 2013). This method centres and projects the PCA using generalised least squares to obtain phylogeny‐independent results.

To estimate evolutionary allometry, we performed multivariate linear regressions with phylogeny, phylogenetic generalised least squares (PGLS), using the ‘procD.pgls’ function from the geomorph package (Adams and Otárola‐Castillo 2013). We thus analysed the relationship between Procrustes coordinates, centroid size and our locomotor groups. We used log centroid size as a conventional proxy for size, as it is calculated on the same structure as the one studied (Hallgrímsson et al. 2019). In addition, we performed linear regressions between our pPCA, the logarithm of the centroid size and the cube root of the logarithm of the mass of our species to assess whether certain principal deformation axes correlate with the overall size of the species.

We investigated the covariation between endocast morphology and pelvis morphology using a two‐block partial least squares phylogenetic analysis (p2BPLS) with the ‘phylo.integration’ tool from the geomorph package (Rohlf and Corti 2000). This method quantifies the degree of phylogenetic morphological integration between two Procrustes forms using partial least squares and a Brownian motion model.

We also performed pairwise analyses on the ProcD.pgls results to identify whether the groups were significantly different. The R code is provided in the supplementary material.

## 3 Results

### 3.1 Phylogenetic Signal

The results of the Kmult tests are all significant with a Kmult value greater than 0.8 (lateral = 1.024, dorsal = 0.940, pelvis = 0.999), indicating a strong influence of phylogeny on the shape of endocast and pelvis.

### 3.2 Shape Analyses

#### 3.2.1 LSO Lateral View pPCA

**Figure 5:**
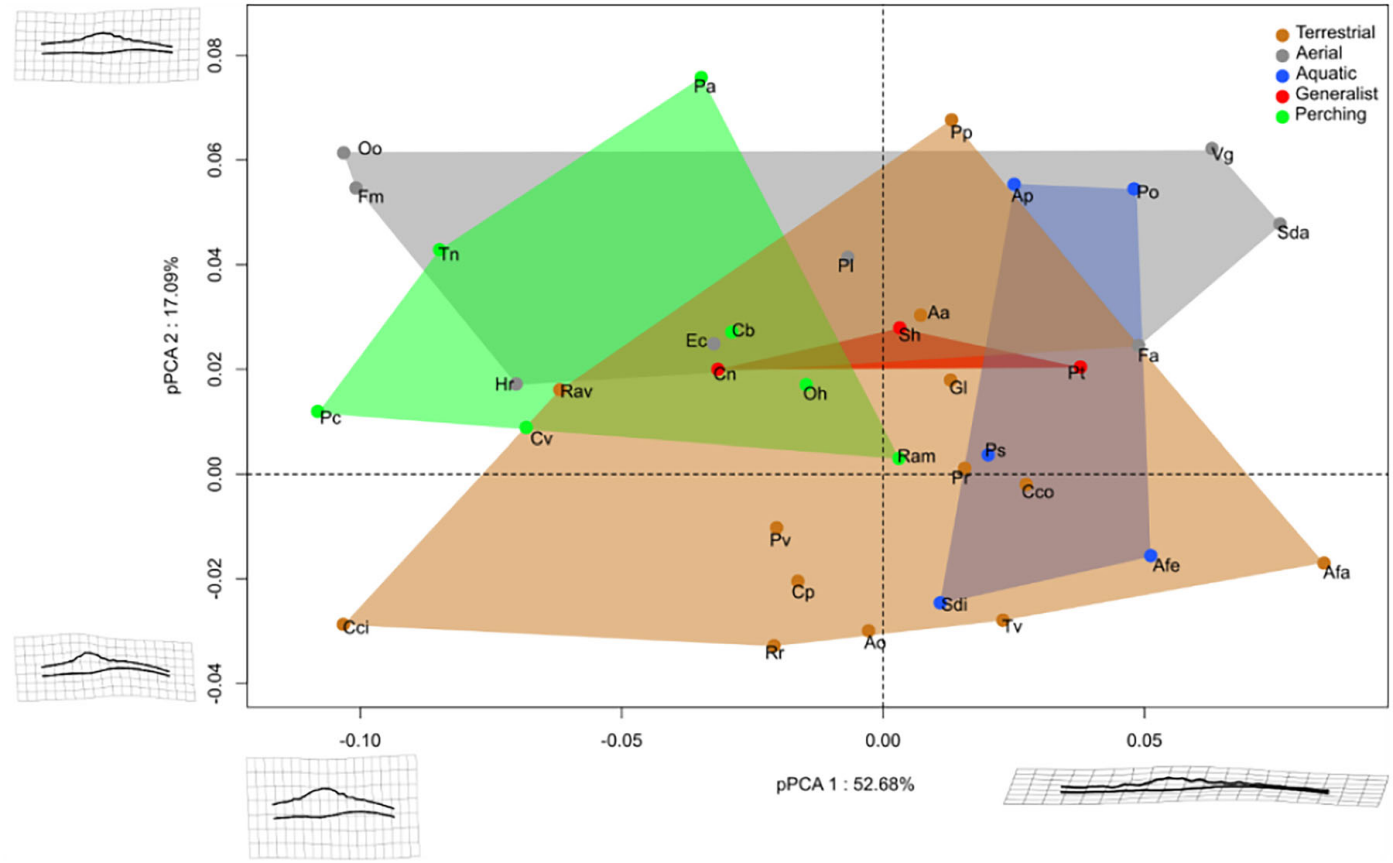
Morphospace formed by pPC1 pPC2 of the LSO in lateral view, shape corresponding to the specific deformations associated with each axis.

The first principal component accounts for 52.68% of the total variation (Figure 5) and corresponds to the craniocaudal length variation. This reflects a relative decrease in the length of the RS compared to the length of the SVC, with more prominent TCs. The second component represents 17.09% and corresponds to the length of the caudal part posterior to the RS.

Terrestrial birds are distributed over almost the entire first and second components. Perching birds are mainly found on the negative side of the first axis and on the positive side of the second axis (short LSO, high RS, short caudal part). The aquatic species are all found only on the positive side of the first axis (long LSO, flat RS, long caudal part). *Pelecanus occidentalis* and *Aptenodytes patagonicus* are positioned toward the positive side of the second axis (short LSO caudal part), while the others are on the negative side. Birds with aerial behaviour are distributed throughout PC1 and are strictly on the positive side (short LSO caudal part) of the second axis. As for the generalist birds, they are positioned at the centre and overlap with all other groups.

The phylogenetic multivariate linear regression on centroid size indicates the presence of significant but low allometry (Shape~log10(Csize): *R*² = 0.13, *p*= 0.032). The analysis detected an effect of ecological categories (Shape~EC: *R*² = 0.48, *p*= 0.005). Our pairwise results indicate that several groups are statistically different from each other (Table 4). Only the Aerials versus Terrestrial, Aquatic versus Perching and Aquatic versus Terrestrial comparisons were significant. We detected allometry on pPCA1 with mass and centroid size (pPCA1~Log10(Mass)^3): *R*² = 0.58, *p* < 0.001; (pPCA1~log10(Csize): *R*² = 0.80, *p* < 0.001). However, given the small size of our groups, these results should be treated with caution.

**Table 4:** Pairwise comparison of ecological groups.

	Lateral	Dorsal	Pelvis
Aerial vs. aquatic	*p* value = 0.089	*p* value = 0.089	*p* value = 0.087
Aerial vs. generalist	*p* value = 0.157	*p* value = 0.138	*p* value = 0.162
Aerial vs. perching	*p* value = 0.065	*p* value = 0.066	*p* value = 0.073
Aerial vs. terrestrial	*p* value = 0.024	*p* value = 0.024	*p* value = 0.027
Aquatic vs. generalist	*p* value = 0.122	*p* value = 0.118	*p* value = 0.124
Aquatic vs. perching	*p* value = 0.016	*p* value = 0.014	*p* value = 0.013
Aquatic vs. terrestrial	*p* value = 0.004	*p* value = 0.004	*p* value = 0.004
Aeneralist vs. perching	*p* value = 0.648	*p* value = 0.531	*p* value = 0.706
Aeneralist vs. terrestrial	*p* value = 0.453	*p* value = 0.365	*p* value = 0.47
Aerching vs. terrestrial	*p* value = 0.114	*p* value = 0.12	*p* value = 0.206

#### 3.2.2 Lubosacral Organ Dorsal View pPCA

**Figure 6:**
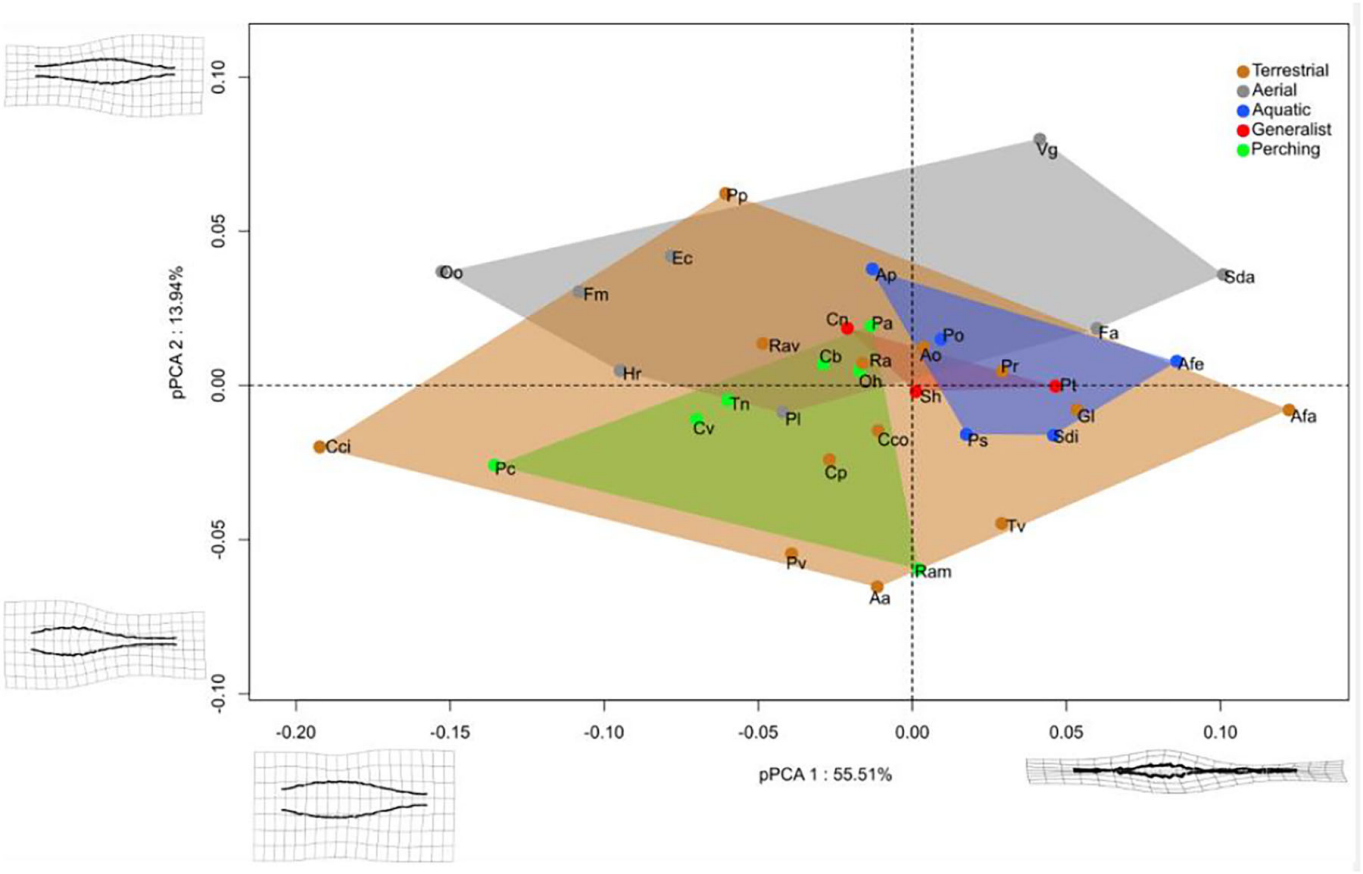
Morphospace formed by pPC1 pPC2 of the LSO in dorsal view, shape corresponding to the specific deformations associated with each axis.

The first component (Figure 6) represents 55.51% of the total variance. This corresponds to the SVC stretching and thinning laterally along its entire length, as well as the RS decreasing in size from the negative to the positive part of the axis. The second component, representing 13.94% of total variance, indicates a more cranial position of the RS within the SVC.

Terrestrial birds are distributed along the first and second axis. Perching birds are mainly found towards the negative side of the first axis. Conversely, aquatic birds are mainly found towards the positive side of the first axis (narrow SVC and RS). The mainly aerial birds are found almost exclusively in the upper part of the second axis (broad SVC). The generalist birds remain in the middle of the graph, halfway between the other groups.

Allometry is present (Shape~log10(Csize): *R*² = 0.15, *p* < 0.006) and associated with ecological categories (Shape~EC: *R*² = 0.41, *p* = 0.006). To validate these observations, pairwise analyses were carried out (see Table 4). Only the comparisons aerials versus terrestrial, aquatic versus perching and aquatic versus terrestrial showed significant differences. However, as the size of the groups is low these results should be interpreted with caution. The pPCA1 also shows a significant degree of allometry (pPCA1~Log10(Mass)^3): *R*² = 0.58 *p* < 0.001; (pPCA1~log10(Csize): *R*² = 0.77 *p* < 0.001).

#### 3.2.3 Pelvis 3D Geometric Morphometric pPCA

The analysis retained four components, which explained respectively 24.71%, 15.62%, 13.79% and 10.94% of the total variance. The first axis mainly expresses variability in the length relative to the width of the pelvis. The second axis mainly describes variation in the relative height of the ischium. Axis 3 mainly describes variability of the location of the acetabulum, and axis 4 variation in the obliquity of the post‐acetabular part.

**Figure 7:**
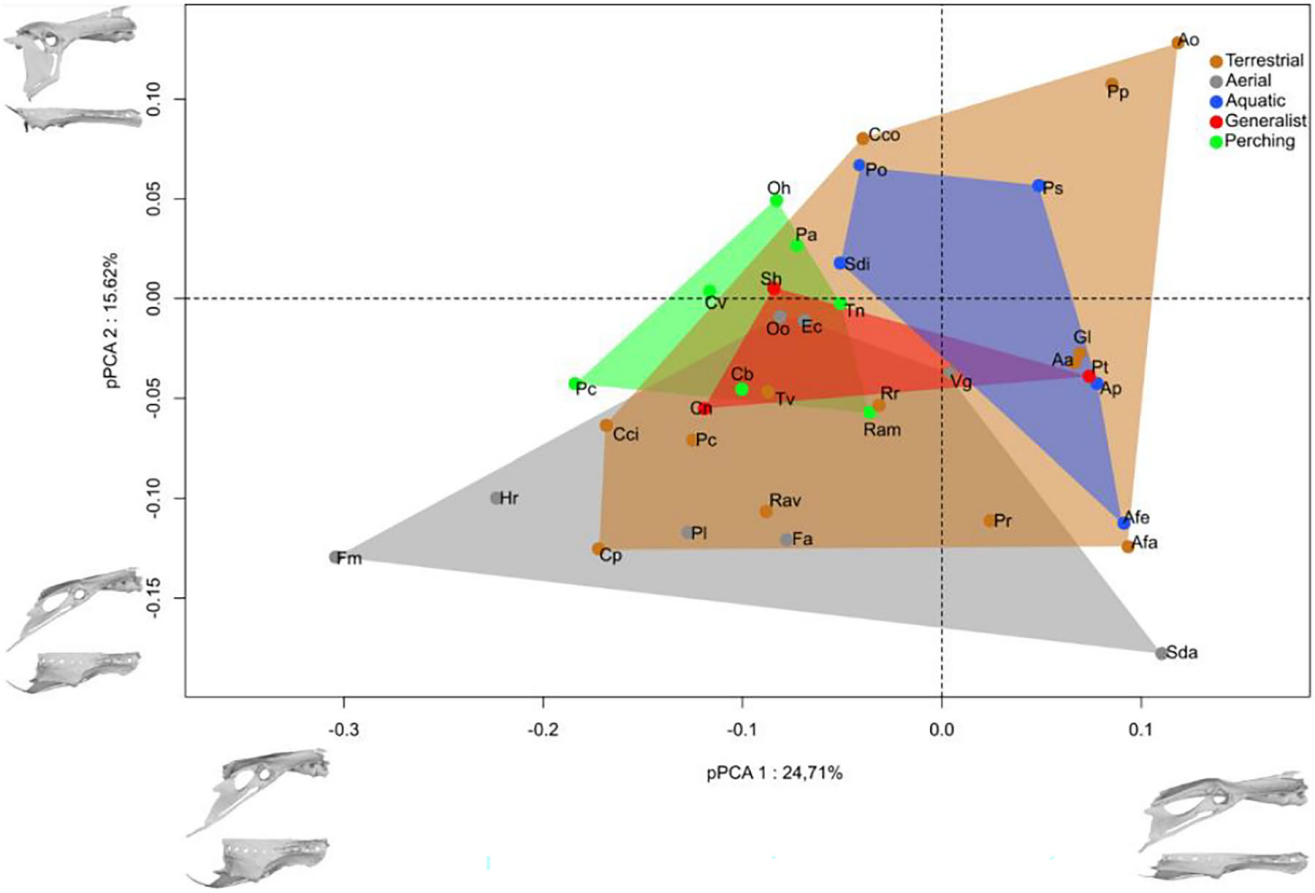
Morphospace formed by pPC1 pPC2 of the pelvis, shape corresponding to the specific deformations associated with each axis.

On the first axis (Figure 7) the synsacrum lengthens in the anteroposterior direction towards the positive side, accompanied by a relative thinning in the lateromedial direction. The relative size of the RS decreases while maintaining its central position in the SVC. The pre‐acetabular iliac wing lengthens anteriorly. The post‐acetabular part migrates strongly posteriorly, with the dorsocaudal end of the iliac crest approaching the posterodorsal end of the ilium. The entire post‐acetabular region becomes more horizontal, with a slight dorsal migration. The pubis becomes proportionally shorter and less curved posteriorly. The acetabulum becomes proportionally larger. Terrestrial and aerial species are distributed along axis 1. Generalist species are confined to the central part of axis 1. Aquatic species are mainly found towards the positive part of axis 1, and have a rather long and narrow pelvis, while perching species are found towards the negative part of axis 1, and have a rather short and wide pelvis.

Along the positive side of axis 2, the pre‐acetabular part lengthens anteriorly, thinning the synsacrum laterally. The relative size of the RS decreases slightly, and it is positioned more caudal in the SVC. The pre‐acetabular iliac wing lengthens anteriorly. The postacetabular part widens dorsoventrally, particularly from the posterodorsal end of the ilium, which migrates further posterodorsally. The acetabulum widens, and the antitrochanter becomes vertical. The pubis becomes proportionally shorter and less curved. The distribution of ecological groups along axis 2 does not show any marked pattern, with the exception of the aerials, which are all located on the negative side. However, *Apteryx owenii* and *Porphyrio poliocephalus*, terrestrial species, have a higher pelvis than the other species, and *Sula dactylatra*, an aerial species, has a rather low pelvis.

**Figure 8:**
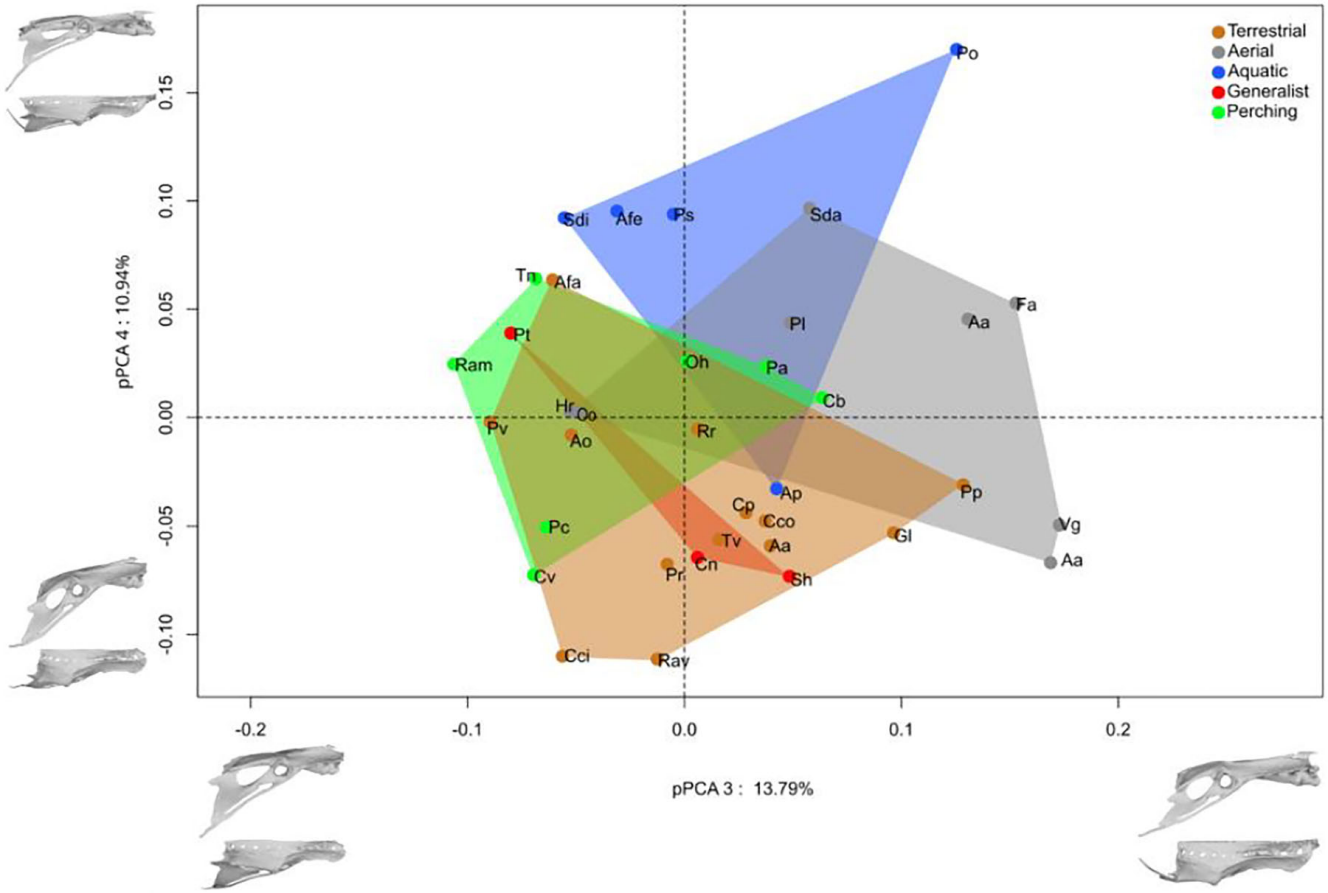
Morphospace formed by pPC3 pPC4 of the pelvis, shape corresponding to the specific deformations associated with each axis.

Along the positive side of axis three (Figure 8), there is an increase in the proportion of the length of the pre‐acetabular part. The synsacrum varies neither in length nor in width in the positive direction along this axis. The RS becomes relatively smaller and moves from a proximal to a central position in the SVC. The tip of the pre‐acetabular part of the ilium migrates slightly posteriorly. The antitrochanteric part migrates ventrally and anteriorly, lengthening the dorsal crest of the ilium. The post‐acetabular part widens posterodorsally. The pubis lengthens and becomes more curved. The distribution of ecological groups along axis 3 shows that the pre‐acetabular part is elongated in aerial species except in *Oceanites oceanicus* and *Hirundo rustica*. The other morphological groups have a fairly central position on this axis.

On the fourth axis, the synsacrum lengthens anteroposteriorly towards the positive side. The relative size of the RS decreases, and its position changes from anterior to central in the SVC. The pre‐acetabular iliac wing shortens considerably posteriorly. The post‐acetabular part tapers posteroventrally, slightly levelling the dorsal crest of the ilium. The pubis becomes proportionally longer and more curved. The distribution of the ecological groups is centred. However, *Aptenodytes patagonicus* is the only aquatic species that shows a tendency towards an oblique post‐acetabular part, whereas the other aquatic species show a post‐acetabular part that is more aligned with the pre‐acetabular part.

Overall, there is an overlap in the pelvic morphological space between the generalist and terrestrial species in our sample. Terrestrial and aerial species overlap in the centre of the space, but show opposite extreme shapes, with a long, narrow and high pelvis in *Apteryx owenii* and *Porphyrio poliocephalus* (terrestrial) and a short, wide pelvis in *Florisuga mellivora* (aerial) and a long, narrow pelvis in *Sula dactylatra* (aerial). While some terrestrial species (*Apteryx owenii*, *Porphyrio poliocephalus*) have a long, high pelvis, others have a short, narrow pelvis. We detected a weak allometry (Shape~log10(Csize): *R*² = 0.12, *p* < 0.001) and a correlation with the ecological categories (Shape~EC: *R*² = 0.26, *p* = 0.002). Pairwise test showed significant differences between aerials versus terrestrial, aquatic versus perching and aquatic versus terrestrial pairs (Table 4). We found the same allometry in pPCA 1 and 2 (pPCA1~Log10(Mass)^3): *R*² = 0.44, *p* < 0.001; pPCA1~log10(Csize): *R*² = 0.56, *p* < 0.001; pPCA2~Log10(Mass)^3): *R*² = 0.02, *p* = 0.46; pPCA2~log10(Csize): *R*² < 0.01, *p* = 0.67).

### 3.3 Ecomorphological Trends

**Figure 9:**
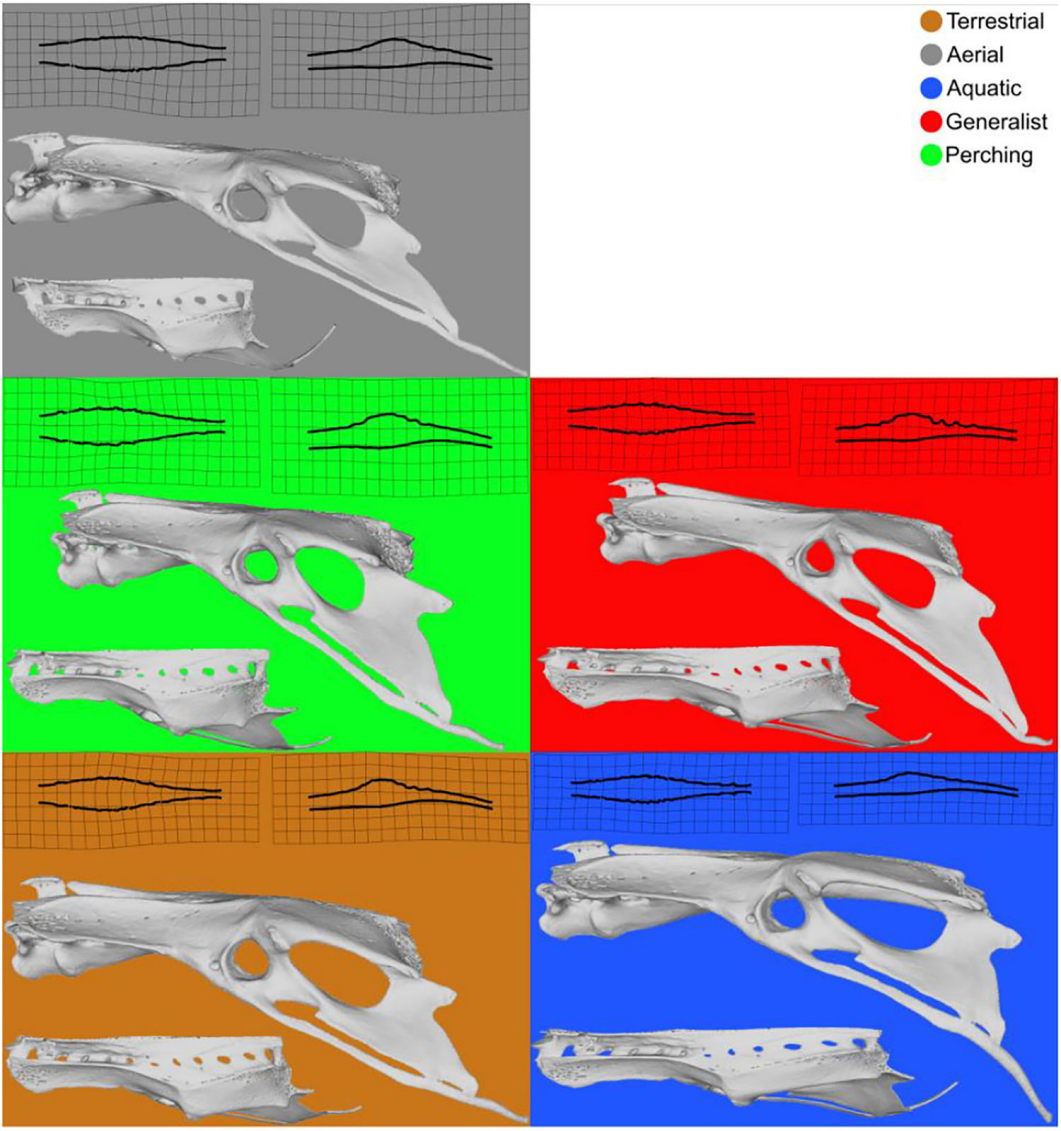
Mean shapes of the synsacral vertebral canal endocast and pelvis for each ecological group.

Eco‐morphological trends are represented in Figure 9 with the mean shape for all ecological groups illustrated.

Aerial birds: The main variation observed in the SVC is that the RS is located centrally within it. The main variation observed in the pelvis is that the preacetabular wing is shorter anteriorly and wider laterally compared to the consensus. The postacetabular portion is shorter in length, but wider laterally than in the other groups. The pubis is proportionally larger and more curved than in the other groups.

Terrestrial birds: The main variation observed in the SVC is that the area containing the RS is slightly more cranially oriented towards the neural canal than in the consensus form and that TCs are more pronounced compared to other groups. The main variations observed in the pelvis is that the synsacrum is shorter and thinner than in the other groups, with the post‐ and preacetabular portions being longer. The dorsal crest of the ilium is proportionally larger than in the aquatic group.

Aquatic birds: The main variation observed in the SVC is a larger SVC and a thinner RS compared to that of other groups. The main variation observed in the pelvis is that the synsacrum is longer and the pelvis is generally thinner than in the consensus. The postacetabular portion is elongated posteriorly, with a dorsal migration of the iliac crest. The acetabulum is wider, and the pubis is more pronounced ventrally.

Perching birds: The main variation observed in the SVC is that it has a short, wide RS compared to other groups. The main variation observed in the pelvis is that the dorsal iliac crest is centrally positioned within the vertebral canal. The synsacrum is slightly shorter anteriorly compared to the aerial group, but wider laterally. The dorsal iliac crest is shorter, and the postacetabular portion is more ventrally inclined.

Generalist birds: The main variation observed in the SVC is that the posterior TCs are visible, as in terrestrial species. The main variation observed in the pelvis is that the dorsal iliac crest is positioned slightly more cranially. The postacetabular portion is shorter, and the pelvis is narrower than in the perching group. The pubis is shorter, and the ischium migrates posteroventrally compared to other groups.

### 3.4 Covariance of the Pelvis Shape With the Lumbosacral Organ Shape

Our results on pPLS1 of the p2BPLS (Figure in Supporting Information S3) are significant with (*p* < 0.001). The first axes of dorsal versus lateral, dorsal versus pelvis and lateral versus pelvis explain 92.57%, 77.69% and 81% of the total covariance of each p2BPLS, respectively. Each of the rPLS values indicates a significant integration between LSO endocast and pelvis morphology (dorsal vs. lateral: rPLS = 0.892; dorsal vs. pelvis: rPLS = 0.756; lateral vs. pelvis: rPLS = 0.799). As the SVC lengthens anteroposteriorly, the RS becomes less imposing and migrates to the centre of the SVC. As the synsacrum lengthens anteroposteriorly and tapers mediolaterally, the pubis shortens.

## 4 Discussion

Our results support and complement the hypothesis proposed by Stanchak et al. (2020). We found that perching birds tend to have larger SVC and RS. The perching lifestyle requires movement in a three‐dimensional environment composed of branches of varying size and flexibility, which implies redundant use of the pelvic limb. It has been demonstrated by experimental modelling (Kamska 2022) that increasing the size of the LSO allows for better reception of locomotor information as well as better protection of the organ itself. It is therefore consistent to find that the LSO, which is a mechanoreceptive organ, is more developed in birds that require a heightened sense of balance. The aerial environment is geometrically less variable than the arboreal environment and does not require pronounced use of the pelvic apparatus. Since the group of aerial birds is polyphyletic and shares this tendency, it could be a convergent trait meeting a functional need for a primarily aerial lifestyle. This position could be associated with better reception of proprioceptive information during flight. The penguin *Aptenodytes patagonicus* shares its SVC shape with primarily aerial birds. Like flying birds, swimming penguins use their wings for propulsion and are subject to the constraints of fluid dynamics (Harada et al. 2021) rather than gravity, which could explain the strong resemblance. Primarily aquatic surface birds are found to have a longer SVC and an RS located in the cranial part of the synsacrum. This could be partly explained by the increase in pelvic length, particularly the postacetabular portion, and by a thinning in the lateromedial direction to maintain the pelvic limbs at waterline level and a greater range of motion to make swimming easier (Frank et al. 2022). The predominantly terrestrial lifestyle does not significantly constrain the shape of the SVC, allowing for greater morphological flexibility. The LSO first emerged in non‐avian theropods and is absent in modern crocodilians (Giffin 1990; Greer et al. 2022). These animals were mainly terrestrial and probably already exhibited diverse and flexible morphologies. During avian evolution, this morphology gradually became more specialised due to constraints related to the main mode of locomotion. Generalist birds are located in the centre of the morphospace and overlap with the other groups, consistent with their locomotor behaviour, as these birds do not have a preferred mode of locomotion.

Our results confirm the existence of allometry affecting the SVC and pelvic morphology. Birds with a lower mass tend to have a proportionally larger RS with a shorter SVC in the anteroposterior direction. This is reflected in pelvic morphology, with a lateromedial and dorsoventral widening of all points near the RS. This could be explained by a physical limit with a minimum critical size beyond which the LSO is no longer efficient or is no longer adequately protected in the SVC. Our results also confirm the existence of significant covariation between the morphology of the SVC, RS, and pelvic morphology. This nonindependence can be explained by the fact that the SVC and RS are located in the synsacrum and therefore follow the morphological variation of the pelvic bones. The morphology of the SVC, associated with that of the pelvis, presents a strong phylogenetic signal. Our results provides new information regarding the link between LSO morphology and phylogeny, while confirming previous results on the pelvis (Anten‐Houston et al. 2017). This result indicates that phylogeny is a major factor determining LSO morphology. This could have future implications for the choice of characters in the phylogenetic reconstruction of fossil birds.

Traces of this organ can be found in the fossil record. The oldest LSO fossil was found in *Ichthyornis* (95–83.5 Ma) (Benito et al. 2022). The first fossil evidence of the existence of LSO was discovered in an indeterminate Ornithurae specimen discovered in 2010, originating from a Maastrichtian site (72.2–66 Ma) (O'Connor and Forster 2010). The most recent example is an early Sphenisciformes fossil from an Eocene site (56–33.9 Ma) (Jadwiszczak et al. 2023). The LSO is thought to be present as early as the Ornithoraces clade, particularly in Enanthionithes and *Confuciusornis* (135–120 Ma) (Mayr et al. 2020). A similar structure was documented by Marsh in 1881 in Stegosaurus (Marsh 1881) and in other dinosaurs (Giffin 1991). It has been interpreted as evidence for the presence of a RS in the lumbosacral region of dinosaurs (Giffin 1991).

## 5 Conclusion

Our data show that the morphology of the SVC is at least partially linked to the locomotor habits of birds. This is particularly true of perching birds, where the RS is much larger than in other groups. Primarily aerial birds, however, seem to have the RS located in the centre of the SVC. The morphology of the SVC is significantly related to overall pelvic morphology. Birds with a large RS tend to have proportionately a short, wide pelvis and a descending post‐acetabular section. Conversely, birds with a smaller RS tend to have a long, narrow pelvis with a horizontal post‐acetabular section. This can be explained by the fact that the SVC is a component of the pelvis, similar to how the morphology of the semicircular canals in birds is constrained by skull and brain size. (Benson et al. 2017). We detected the presence of an evolutionary allometry in the morphology of the SVC and pelvis, indicating that bird size has an impact on the morphology of the LSO. Finally, the morphology of the SVC also shows a strong phylogenetic signal. This tends to indicate that the LSO is constrained and that the general morphology of the SVC has been maintained during evolution. While our results reinforce previous hypotheses and expand our knowledge of this subject, our study has some limitations, including the sample size and the use of 2D morphometric approaches. We therefore encourage future studies to carry out comparative anatomical descriptions between several species, as well as research in the fossil record. This would increase our overall knowledge of the LSO, and enable us to study it more effectively.

## Author Contributions

**Idriss Pelletan:** conceptualisation, writing – original draft, writing – review and editing, software, methodology, formal analysis, investigation, validation. **Raphaël Cornette:** writing – review and editing, conceptualisation, methodology, supervision, formal analysis. **Anick Abourachid:** conceptualisation, writing – review and editing, validation, supervision, funding acquisition.

## 1 Peer Review

The peer review history for this article is available at https://www.webofscience.com/api/gateway/wos/peer-review/10.1002/jmor.70073.

## Supporting information

code LSO.

Résumé.

Suplementary data.

## Data Availability

The data that support the findings of this study are available on request from the corresponding author. The data are not publicly available due to privacy or ethical restrictions.
