# Supplementary material for: Ecomorphological Analysis of the Bird Lumbosacral Organ in an Evolutionary Context
Source: J Morphol. 2025 Aug 23;286(8):e70073. doi: 10.1002/jmor.70073 (PMC12374226; doi:10.1002/jmor.70073)
Supplement: Supplementary file 2 — Résumé. [file JMOR-286-e70073-s002.docx]

Les oiseaux possèdent leur propre organe d'équilibre, l'organe lombo-sacré (LSO), qui est unique parmi les animaux existants. Situé dans le synsacrum, dans la région lombo-sacrée, le LSO entoure la moelle épinière et déforme le canal neural, laissant des traces identifiables de sa taille et de sa forme sur l'endocaste. Au moment où nous écrivons ces lignes, le LSO suscite encore de nombreuses interrogations quant à sa fonction et à ses diverses implications dans la biologie des oiseaux. Dans cet article, nous cherchons à savoir si la forme de l'endocaste du canal neural dans le synsacrum formé par le LSO est liée aux habitudes locomotrices, à la morphologie pelvienne et à la phylogénie.

Pour effectuer nos recherches, nous avons utilisé des outils de morphométrie géométrique 2D et 3D afin de caractériser la forme de l'endocaste neural du canal synsacrum. Cette forme a été mise en relation avec le comportement locomoteur ainsi qu’avec les morphologies pelviennes. Nous avons également quantifié le signal phylogénétique contenu dans la morphologie de l'endocaste du canal neural et du bassin afin de déterminer si la phylogénie a un impact significatif sur la morphologie.

Nos résultats suggèrent que l'endocaste du canal neural est façonné par le LSO, en particulier chez les oiseaux percheurs. Nous montrons également que la morphologie pelvienne covarie de manière significative avec la morphologie du canal neural. Un LSO proportionnellement plus grand correspond à un bassin plus court et plus large, tandis qu'un LSO plus petit correspond à un bassin plus long et plus fin. Enfin, nous avons montré la présence d'un signal phylogénétique fort dans les canaux neuraux.
