# Supplementary material for: Ecomorphological Analysis of the Bird Lumbosacral Organ in an Evolutionary Context
Source: J Morphol. 2025 Aug 23;286(8):e70073. doi: 10.1002/jmor.70073 (PMC12374226; doi:10.1002/jmor.70073)
Supplement: Supplementary file 3 [file JMOR-286-e70073-s001.odt]

| Species | Common name |  |  | Species | Common name |  |  |
| --- | --- | --- | --- | --- | --- | --- | --- |
| Elanus caeruleus | Black-winged kite | Natural History Museum (London) | NHMUK_1850.8.15.159 | Opisthocomus hoazin | Hoatzin | Natural History Museum (London) | NHMUK_S1961.6.1 |
| Florisuga mellivora | White-necked jacobin | Yale University | YPM_101545 | Probosciger aterrimus | Palm cockatoo | Natural History Museum (London) | NHMUK_S2006.15.5 |
| Fregata aquila | Ascension frigatebird | Natural History Museum (London) | NHMUK_1890.11.3.3 | Pycnonotus cafer | Red-vented bulbul | Natural History Museum (London) | NHMUK_S1986.75.18 |
| Hirundo rustica | Barn swallow | Natural History Museum (London) | NHMUK_A2007.15.5 | Ramphastos ambiguus | Yellow-throated toucan | Natural History Museum (London) | HMUK_S2002.21 |
| Oceanites oceanicus | Wilson's storm petrel | Natural History Museum (London) | NHMUK_S_1957.11.2 | Tockus nasutus | African grey hornbill | Natural History Museum (London) | NHMUK_S_1989.2.2 |
| Phaeton lepturus | White-tailed tropicbird | Natural History Museum (London) | NHMUK_1876.3.16.3 | Anser fabalis | Taiga bean goose | Natural History Museum (London) | HMUK_1895.2.6.9 |
| Sula dactylatra | Masked booby | Natural History Museum (London) | NHMUK1890.11.3.10 | Ardea alba | Great egret | University of Cambridge | UMZC_338H |
| Vultur gryphus | Andean condor | Natural History Museum (London) | NHMUK_S1955.5.11 | Calandrella cinerea | Red-capped lark | Natural History Museum (London) | NHMUK_S1961.13 |
| Pelecanus occidentalis | Brown pelican | Natural History Museum (London) | NHMUK_S1973.66.16 | Columba palumbus | Common wood pigeon | University of Cambridge | UMZC_409A |
| Spatula discors | Blue-winged teal | Natural History Museum (London) | NHMUK_S2001.41.5 | Coturnix coturnix | Common quail | Muséum national d’Histoire naturelle (Paris | Ci06 |
| Aptenodytes patagonicus | King penguin | University of Cambridge | UMZC_184D | Grus leucogeranus | Siberian crane | University of Cambridge | UMZC_344D |
| Aythya ferina | Common pochard | Natural History Museum (London) | NMHUK_1851.12 | Phoenicopterus ruber | American flamingo | University of Cambridge | UMZC_346B |
| Podica senegalensis | African finfoot | University of Cambridge | UMZC_209A | Picus viridis | European green woodpecker | Natural History Museum (London) | NHMUK_S1982.5.1 |
| Chroicocephalus novaehollandiae | Silver gull | University of Cambridge | UMZC_274.C | Porphyrio poliocephalus | Grey-headed swamphen | Natural History Museum (London) | NHMUK_S1952 |
| Puffinus tenuirostris | Short-tailed shearwater | Natural History Museum (London) | NHMUK_1850.8.15.152 | Recurvirostra avosetta | Pied avocet | Natural History Museum (London) | NHMUK_S1962.10.1 |
| Strigops habroptilus | Kākāpō | Natural History Museum (London) | NHMUK_1881.1.17.71 | Rollulus rouloul | Crested partridge | Natural History Museum (London) | NHMUK_1871.7.20.87 |
| Calyptomena viridis | Green broadbill | Natural History Museum (London) | NHMUK_S1969.1.70 | Turnix varius | Painted buttonquail | Natural History Museum (London) | NHMUK_S1952.2.142 |
| Coracias benghalensis | Indian roller | Natural History Museum (London) | NHMUK_S1987.19.15 | Apteryx owenii | Little spotted kiwi | University of Cambridge | UMZC_378iii |

Supplementary table 1: List of the specimens use in this study

Fig sup 1 : PLS1 Dorsal vs Lateral

Fig sup 2 : PLS1 Dorsal vs Pelvis

Fig sup 3 : PLS1 Dorsal vs pelvis
